# A roadmap for the development and evaluation of the eHealthResp online course

**DOI:** 10.1177/20552076221089088

**Published:** 2022-03-24

**Authors:** Marta Estrela, Tânia Magalhães Silva, Ana Margarida Pisco Almeida, Carlos Regueira, Maruxa Zapata-Cachafeiro, Adolfo Figueiras, Fátima Roque, Maria Teresa Herdeiro

**Affiliations:** 1 451098 iBiMED – Institute of Biomedicine, Department of Medical Sciences, University of Aveiro, Aveiro, Portugal; 2 Department of Communication and Art/DigiMedia, University of Aveiro, Aveiro, Portugal; 3 Department of Preventive Medicine and Public Health, University of Santiago de Compostela, 15702 Santiago de Compostela, Spain; 4 Consortium for Biomedical Research in Epidemiology and Public Health (CIBER Epidemiology and Public Health - CIBERESP), Santiago de Compostela, Spain; 5 Health Research Institute of Santiago de Compostela (IDIS), University of Santiago de Compostela, Santiago de Compostela, Spain; 6 Research Unit for Inland Development, 70920 Guarda Polytechnic Institute (UDI-IPG), Guarda, Portugal; 7 Health Sciences Research Center, University of Beira Interior (CICS-UBI), Covilhã, Portugal

**Keywords:** Usability, health professionals, online course, respiratory infections, e-learning, website

## Abstract

**Background:**

Inappropriate antibiotic use constitutes one of the most concerning public health issues, being one of the main causes of antibiotic resistance. Hence, to tackle this issue, it is important to encourage the development of educational interventions for health practitioners, namely by using digital health tools. This study focuses on the description of the development and validation process of the eHealthResp online course, a web platform directed to physicians and pharmacists, with the overall goal of improving antibiotic use for respiratory tract infections, along with the assessment of its usability.

**Methods:**

The eHealthResp platform and the courses, developed with a user-centered design and based on Wordpress and MySQL, were based on a previously developed online course. A questionnaire to assess the usability was distributed among physicians (n = 6) and pharmacists (n = 6). Based on the obtained results, statistical analyses were conducted to calculate the usability score and appraise the design of the online course, as well as to compare the overall scores attributed by both groups. Further qualitative comments provided by the participants have also been analyzed.

**Results:**

The eHealthResp contains two online courses directed to physicians and pharmacists aiming to aid in the management of respiratory tract infections. The average usability score of the eHealthResp online courses for physicians and pharmacists was of 78.33 (±11.57, 95%CI), and 83.75 (±15.90, 95%CI), respectively. Qualitative feedback emphasized the usefulness of the course, including overall positive reviews regarding user-friendliness and consistency.

**Conclusions:**

This study led us to conclude that the eHealthResp online course is not recognized as a complex web platform, as both qualitative and quantitative feedback obtained were globally positive.

## Background

Antibiotic resistance is considered one of the major Public Health threats worldwide, with inappropriate use of antibiotics being one of the main concerns, especially for respiratory tract infections.^1–4^ Considering: (i) that respiratory diseases are one of the leading causes of death and disability, (ii) the high incidence of respiratory tract infections, and (iii) the widespread overuse of antibiotics for these diseases, interventions to improve antibiotic use constitute an essential approach.^3–5^ However, the effectiveness of antimicrobial stewardship interventions strongly depends on an adequate design, tailored to each setting.^6–8^

Allying digital health tools to educational interventions for health practitioners can significantly improve healthcare quality,^9–11^ going from the reduction of medication errors^12–14^ to the improvement of antibiotic prescription quality.^15,16^ e-Health instruments, especially clinical decision support systems (CDSS), comprise a multiplicity of tools that aid in clinical decision making, thus saving time needed to strengthen the relationship with patients and facilitating the act of providing care.^13,17–19^ Hence, as increasingly new information on antibiotic use emerges, educating and informing both patients and health professionals becomes highly essential to enhance clinical practices, ensuring they are up-to-date.^20^

Our research group developed eHealthResp,^21^ a digital platform comprising two online courses, one directed to primary care physicians and the other one to community pharmacists. Both courses address respiratory infections’ management, with the goal of improving healthcare quality,^9–11^ and, ultimately, promoting adequate antibiotic use,^12–16^ specifically for respiratory tract infections.

To improve the overall design and ensure the adequacy and user-friendliness of the eHealthResp online courses, the evaluation of their usability constitutes a critical step for the assessment of digital applications in human health.^22^ Thus, the main goal of this study is to provide a description of the development and validation process of the eHealthResp online course, having as the main outcome the assessment of its usability by using the System Usability Scale (SUS).^23,24^ Furthermore, as the web platform in which both online courses are embedded is the same, this study aims to compare the results obtained between the usability scores provided by physicians and pharmacists.

## Methods

### eHealthResp project and website

The eHealthResp website is part of a research project that comprises an educational intervention designed for primary care physicians and community pharmacists, which will be conducted through a cluster randomized controlled trial on the geographical area of Portugal's Center Regional Health Administration (ARS-C). This intervention consists of an online course and a mobile app composed by several algorithms for the management of respiratory tract infections in adults, serving as a useful aid to the clinical decision process.

The eHealthResp platform and the courses, were developed with a user-centered design, and were based on a previously developed online course.^25^ After a thorough bibliographic review and testing of different platforms, the re-organization of content and navigation structure has been conducted. The website was then developed on a Wordpress and MySQL based system, with Elementor and LifterLMS, a learning management system, as main plugins.

### Web platform and course overview

The eHealthResp is a Wordpress-based web platform that contains two self-paced online courses directed to physicians and pharmacists aiming to aid in the management of respiratory tract infections. Additionally, the webpage serves as a host to the eHealthResp project's information, including a contacts section, a publications section, and a page for the download of the mobile app *(see supplementary material S1)*.

The physicians’ course contains four sections, consisting of: 1) an introduction to the online course's contents and a brief overview regarding respiratory tract infections; 2) six modules on specific respiratory tract infections (namely i) acute otitis media, ii) acute rhinosinusitis, iii) acute pharyngitis, iv) acute bronchitis, v) community-acquired pneumonia, with an additional module for vi) differential diagnosis of COVID-19); 3) four clinical cases; 4) satisfaction questionnaire and course completion page. Similarly, the pharmacists’ online course contains the same structure, apart from having only three modules (specifically i) common cold and flu, ii) acute rhinosinusitis, acute pharyngitis, and acute bronchitis and iii) acting protocol), instead of six. Each group of health professionals has access to their reserved area.

### Course and module structure

The module pages consist of a slideshow section, in which the user can navigate freely through the presentation. At the bottom of the slideshow section, the page presents a “Mark as complete” button, to register the module as completed thus granting access to the next module, and a “Download” button, which allows the users to save the presentation as a PDF file to their devices and access them offline. Furthermore, these pages also include two navigation buttons, to return to the previous module or to advance towards the next.

### Content validation

The eHealthResp online courses’ contents have been subjected to content validation through a Delphi Method approach.^26^ For this study, several experts have been invited to help to improve both online courses’ contents, providing feedback regarding several clinical cases which were further included in the presentations and clinical cases sections. Besides the content validation, and since the eHealthResp platform has been developed with a user-centered design, two usability studies have also been conducted.

### Usability testing

Six physicians and six pharmacists^24^ were recruited through a convenience sample to participate in a study aiming to validate the usability of the online course strictly directed to physicians and pharmacists, respectively. The participants were invited to participate in the study and asked to explore the site contents, with a special focus on the usability of the website.^27,28^ To provide them with access to the restricted area, the website's URL was sent by e-mail, along with an individual username and password and requesting participants to fully explore the website and the online course. These credentials granted access to the course contents, providing them with full autonomy to explore the website.

Along with the access credentials sent by e-mail, a hyperlink to the usability questionnaire was also sent to each participant. This questionnaire was composed by ten mandatory closed-ended questions, based on the System Usability Scale, and an optional comment box, in which participants were able to provide comments about their user experience. Participants were given around two weeks to fully explore the website pages and to complete the online course.

In accordance with the General Data Protection Regulation (GDPR), participants provided their informed consent for the website credentials and questionnaire to be sent to their e-mails. Furthermore, each participant was informed about the objectives of this study and freely consented to participate in this study, providing their consent when answering the questionnaire.

#### System usability scale

The System Usability Scale (SUS) consists of a group of ten questions, in which participants should provide an answer based on a 5-point Likert scale numbered from 1 (“Strongly disagree”) to 5 (“Strongly agree”).^24,29–31^ To calculate the usability score for each participant, odd-numbered questions (SOQ) scores and even-numbered questions (SEQ) scores were combined to obtain a 100-point scale.^23,32^

#### Questionnaire's results analysis

Descriptive statistical analyses were conducted to evaluate the usability of the online course. To ensure the adequacy of the scale, internal reliability statistical tests were performed through the calculation of Cronbach's alpha.^24,29–31^ As the variables did not follow a normal distribution, non-parametric tests were conducted. Hence, the differences between physicians and pharmacists were evaluated using the Mann–Whitney U test The outcomes were established as statistically significant at p < 0.05. Moreover, the research team analyzed the qualitative data obtained to better understand the final feedback about the course.

## Results

### Content validation and usability testing

After conducting the usability study with both physicians and pharmacists, the average score attributed by each group was of 78.33 (±11.57, 95%CI), and 83.75 (±15.90, 95%CI), respectively. The table 1 presented below compares the overall perception between physicians and pharmacists:

**Table 1.** Comparison between physicians’ and pharmacists’ usability evaluation.

**Item**	**Median (PCT25, PCT75)**		**Mann-Whitney (p-value)**
	**Physicians**	**Pharmacists**	
**1. I think that I would like to use this system frequently.**	3.50 (2.75, 4.00)	4.50 (3.50, 5.00)	0.13
**2. I found the system unnecessarily complex.**	2.00 (1.00, 2.50)	1.50 (1.00, 2.25)	0.70
**3. I thought the system was easy to use.**	5.00 (4.75, 5.00)	5.00 (3.75, 5.00)	0.59
**4. I think that I would need the support of a technical person to be able to use this system.**	1.00 (1.00, 2.00)	1.00 (1.00, 2.00)	0.82
**5. I found the various functions in this system were well integrated.**	3.50 (3.00, 4.00)	5.00 (4.00, 5.00)	0.02
**6. I thought there was too much inconsistency in this system.**	2.50 (2.00, 3.25)	1.50 (1.00, 3.25)	0.31
**7. I would imagine that most people would learn to use this system very quickly.**	4.50 (3.75, 5.00)	5.00 (3.75, 5.00)	0.70
**8. I found the system very cumbersome to use.**	1.00 (1.00, 2.50)	1.00 (1.00, 2.25)	1.00
**9. I felt very confident using the system.**	5.00 (4.75, 5.00)	4.50 (4.00, 5.00)	0.39
**10. I needed to learn a lot of things before I could get going with this system.**	1.00 (1.00, 1.75)	2.00 (1.00, 3.50)	0.39
**Total score**	85.00 (65.63, 88.13)	91.25 (64.38, 98.13)	0.49

As observed in table 1, most of the scores between physicians and pharmacists followed a similar distribution. However, a statistically significant difference was detected regarding the integration of the website's functions, where physicians tended to attribute a lower score than pharmacists. Nevertheless, the average overall score between physicians and pharmacists has differed less than 5 points.

### Qualitative feedback

To complement the quantitative feedback obtained through the SUS's results, a comment box was included on the usability questionnaire sent to physicians and pharmacists. Though not all participants provided further comments on the eHealthResp online course, those who did have highlighted the eHealthResp online course usefulness and user-friendliness:

*“I found the course very objective and practical. The search and navigability are not complex allowing good accessibility to the content.” – Pharmacist 1*

“[The eHealthResp online course] is easy to use and very objective” – Physician 1

“Very educational. Clearly presented cases, without any doubts. Very useful for testing the quick thinking of diagnosis and treatment” – Physician 2

Still, some physicians have also suggested some improvements in the online course's contents:

“I suggest some corrections in the course contents, namely in the topic otitis media and acute pharyngitis.” – Physician 3

“Since the course is aimed at physicians, scientific language described in each pathology should be improve and adapted, as well as in the description of clinical cases.” – Physician 4

### eHealthResp development roadmap

**Figure 1.**
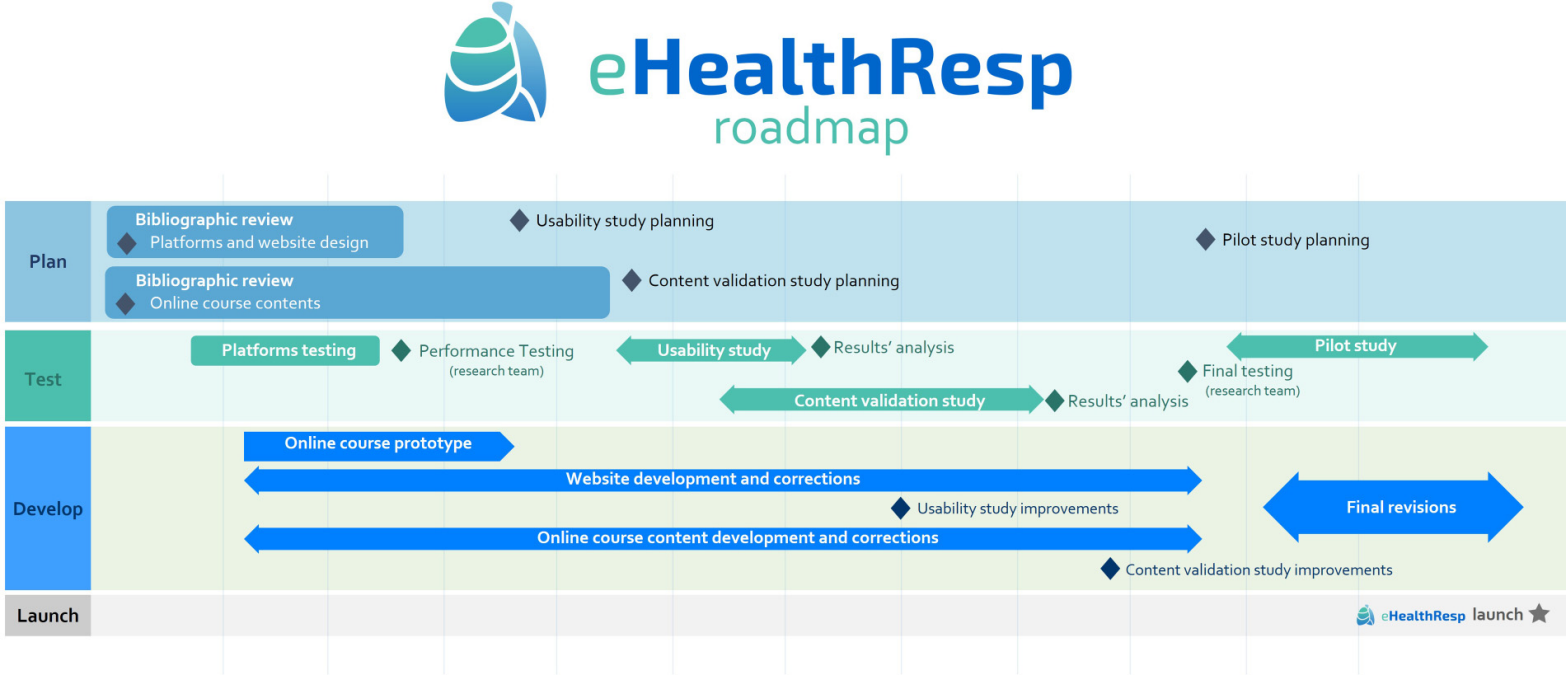
Ehealthresp development roadmap.

After the content validation and usability testing, the eHealthResp online course and contents have been readjusted by the research team. The Figure 1 illustrates a roadmap for the eHealthResp online course development, from its first development stages until its launch.

## Discussion

By following a user-centered design, the eHealthResp online course demanded a close interaction with the end-user throughout the development process. This iterative process, conducted through the validation of both the online courses’ contents^26^ and the usability of the web platform, constitutes one of the main strengths of this educational intervention. Overall, considering the highly positive feedback provided by the participants, the results obtained with the usability study reveal that the eHealthResp web platform does not show signs of inconsistencies, and is not perceived as a complex platform.

As most of the positive items have received a score above four, and most of the negative items had an average score of 2 or below, these outcomes reflect the user-friendliness of the online course, being in line with other usability studies for e-learning tools, especially with physicians, in which the usability score obtained has shown to be similar.^31,33,34^

When comparing to the results obtained for the online course for pharmacists, only the question related to function integration has had a statistically significant lower score. Yet, the overall feedback on this statement has remained positive on both groups. Hence, the similarity between results and generally good scores gives us a sense of consistency and quality of both online courses and the web platform. As the reports provided by health professionals were positive towards eHealthResp and other systems, the notion that these tools can strongly improve clinical practice is here reinforced.^35–39^

Moreover, it is important to note that participants using a tablet or cell phone to explore the eHealthResp website and online courses provided an average score significantly lower (> 20 points) than the scores provided by those using a computer/laptop. Despite these differences are in agreement with the literature,^40^ a possible explanation might be associated to the fact that most of the educational content is available in a slideshow mode, which might be more adequate for a computer/laptop screen. However, issues regarding scale and ease in navigation through the website might also contribute to the observed differences. Nevertheless, to tackle these difficulties, a “Download” button has been included, so contents can be easily downloaded as a PDF file, thus allowing to scale the contents easily, offline, and outside the browser.

Yet, although the SUS has several strengths, such as content validity and reliability,^23,41,42^ and allows for a simple usability assessment, it only provides quantitative feedback. Hence, to tackle this lack of specificity, a commentary section has been added to the questionnaire, so participants could provide their qualitative feedback if they deemed necessary. Despite of being an optional evaluation parameter, five out of six physicians and one pharmacist have left some suggestions, most of them being related with technical corrections to the online course contents. However, when considering the comments provided by participants regarding the web platform *per se*, only positive feedback has been obtained, with emphasis on eHealthResp online course's easiness to use, overall aspect, and usefulness, which reinforces its viability.

Even though the end-goal of this study was to evaluate the usability of the eHealthResp online course, the technical comments on the contents were also taken into account, complementing the previously content validation study conducted by our group,^26^ and improving its overall quality.

## Conclusions

The usability evaluation of the eHealthResp presented positive overall scores in terms of user-friendliness, complexity, and consistency. The eHealthResp online course aims to aid health practitioners to manage respiratory tract infections, and this study has allowed to obtain qualitative feedback from possible future users of the online course, which is currently being prepared for a pilot study involving a group of health professionals.

Usability is a very important dimension when developing digital educational contents. The validation of the online course eHealthResp, both in terms of its contents and usability, will support in the improvement of the educational intervention that will cover all primary care physicians and community pharmacists in the Center Region of Portugal, belonging to the ARS-C, as a cluster randomized controlled trial. We believe that this study may be an important description of the different phases that take place throughout an online course design and development, and thus hope to serve as a model to future educational interventions – not only for antibiotic resistances and respiratory diseases management but for clinical practice in general.

## Supplemental Material

sj-pptx-1-dhj-10.1177_20552076221089088 - Supplemental material for A roadmap for the development and evaluation of the eHealthResp online course Click here for additional data file. Supplemental material, sj-pptx-1-dhj-10.1177_20552076221089088 for A roadmap for the development and evaluation of the eHealthResp online course by Marta Estrela, Tânia Magalhães Silva, Ana Margarida Pisco Almeida, Carlos Regueira, Maruxa Zapata-Cachafeiro, Adolfo Figueiras, Fátima Roque and Maria Teresa Herdeiro in Digital Health

## References

[1] Cassini A Högberg LD Plachouras D , et al. Attributable deaths and disability-adjusted life-years caused by infections with antibiotic-resistant bacteria in the EU and the European Economic Area in 2015: a population-level modelling analysis. Lancet Infect Dis 2019; 19: 56–66. 30409683 10.1016/S1473-3099(18)30605-4 PMC6300481

[2] Deuster S Roten I Muehlebach S . Implementation of treatment guidelines to support judicious use of antibiotic therapy. J Clin Pharm Ther 2010; 35: 71–78. 20175814 10.1111/j.1365-2710.2009.01045.x

[3] Forum of International Respiratory Societies, Respiratory diseases in the world Realities of Today-Opportunities for Tomorrow, 1st ed., European Respiratory Society, Sheffield, 2013. https://www.theunion.org/what-we-do/publications/technical/english/FIRS_report_for_web.pdf.

[4] Pattemore PK Jennings LC . Epidemiology of respiratory infections. In: Pediatric Respiratory Medicine. Netherlands: Elsevier, 2008, pp.435–452. 10.1016/B978-032304048-8.50035-9.

[5] Shively NR Buehrle DJ Clancy CJ , et al. Prevalence of inappropriate antibiotic prescribing in primary care clinics within a veterans affairs health care system. Antimicrob Agents Chemother 2018; 62: e00337-18. 10.1128/AAC.00337-18 PMC6105840 29967028

[6] Roque F Teixeira-Rodrigues A Breitenfeld L , et al. Decreasing antibiotic use through a joint intervention targeting physicians and pharmacists. Future Microbiol 2016; 11: 877–886. 27415585 10.2217/fmb-2016-0010

[7] Lopez-Vazquez P Vazquez-Lago JM Figueiras A . Misprescription of antibiotics in primary care: a critical systematic review of its determinants. J Eval Clin Pract 2012; 18: 473–484. 21210896 10.1111/j.1365-2753.2010.01610.x

[8] Arnold S Straus S . Interventions to improve antibiotic prescribing practices in ambulatory care. Evidence-Based Child Heal A Cochrane Rev J 2006; 1: 623–690. 10.1002/ebch.28 PMC7163637 32313517

[9] Roque F Herdeiro MT Soares S , et al. Educational interventions to improve prescription and dispensing of antibiotics: a systematic review. BMC Public Health 2014; 14: 1276. 10.1186/1471-2458-14-1276 PMC4302109 25511932

[10] Gulliford MC Prevost AT Charlton J , et al. Effectiveness and safety of electronically delivered prescribing feedback and decision support on antibiotic use for respiratory illness in primary care: REDUCE cluster randomised trial. Br Med J 2019; 364: l236. 30755451 10.1136/bmj.l236 PMC6371944

[11] Carvalho É Estrela M Zapata-Cachafeiro M , et al. E-Health tools to improve antibiotic use and resistances: a systematic review. Antibiotics 2020; 9: 05. 10.3390/antibiotics9080505 PMC7460242 32806583

[12] Velickovski F Ceccaroni L Roca J , et al. Clinical decision support systems (CDSS) for preventive management of COPD patients. J Transl Med 2014; 12: S9. 25471545 10.1186/1479-5876-12-S2-S9 PMC4255917

[13] António Ferreira Rodrigues Nogueira M Tygesen H Eriksson H , et al. Clinical decision support system (CDSS) – effects on care quality. Int J Health Care Qual Assur 2014; 27: 707–718. 25417376 10.1108/ijhcqa-01-2014-0010

[14] Litvin CB Ornstein SM Wessell AM , et al. Use of an electronic health record clinical decision support tool to improve antibiotic prescribing for acute respiratory infections: the ABX-TRIP study. J Gen Intern Med 2013; 28: 810–816. 23117955 10.1007/s11606-012-2267-2 PMC3663943

[15] McGinn TG McCullagh L Kannry J , et al. Efficacy of an evidence-based clinical decision support in primary care practices: a randomized clinical trial. JAMA Intern Med 2013; 173: 1584–1591. 23896675 10.1001/jamainternmed.2013.8980

[16] Figueiras A López-Vázquez P Gonzalez-Gonzalez C , et al. Impact of a multifaceted intervention to improve antibiotic prescribing: a pragmatic cluster-randomised controlled trial. Antimicrob Resist Infect Control 2020; 9: 1–12. 33287881 10.1186/s13756-020-00857-9 PMC7722452

[17] Carracedo-Martinez E Gonzalez-Gonzalez C Teixeira-Rodrigues A , et al. Galician pharmacoepidemiology research group, computerized clinical decision support systems and antibiotic prescribing: a systematic review and meta-analysis. Clin Ther 2019; 41: 552–581. 30826093 10.1016/j.clinthera.2019.01.018

[18] Kawamoto K Houlihan CA Balas EA , et al. Improving clinical practice using clinical decision support systems: a systematic review of trials to identify features critical to success. Br Med J 2005; 330: 65. 15767266 10.1136/bmj.38398.500764.8F PMC555881

[19] Kannry J McCullagh L Kushniruk A , et al. A framework for usable and effective clinical decision support: experience from the iCPR randomized clinical trial. EGEMS (Generating Evid Methods to Improv Patient Outcomes) 2017; 3: 10. 10.13063/2327-9214.1150 PMC4537146 26290888

[20] Bremmer DN Trienski TL Walsh TL , et al. Role of technology in antimicrobial stewardship. Med Clin North Am 2018; 102: 955–963. 30126584 10.1016/j.mcna.2018.05.007

[21] Herdeiro MT Roque F Figueiras A . eHealthResp – Inspirar conhecimento; 2021. https://ehealthresp.web.ua.pt/.

[22] Maramba I Chatterjee A Newman C . Methods of usability testing in the development of eHealth applications: a scoping review. Int J Med Inform 2019; 126: 95–104. 31029270 10.1016/j.ijmedinf.2019.03.018

[23] Martins AI Rosa AF Queirós A , et al. European Portuguese validation of the system usability scale (SUS). Netherlands: Elsevier B.V., 2015. 10.1016/j.procs.2015.09.273.

[24] Moura J Estrela M Almeida AM , et al. A usability study of Pharmacists’ perceptions toward an online course for respiratory infections and antibiotic use. Procedia Comput Sci 2021; 181: 269–276.

[25] Zapata Cachafeiro M . Evaluación de la efectividad de una intervención educativa en farmacéuticos comunitarios para mejorar la atención farmacéutica en gripe, catarro y otras infecciones de las vías respiratorias altas; 2019. http://hdl.handle.net/10347/18328 (accessed July 9, 2021).

[26] Estrela M Roque F Silva TM , et al. Validation of the eHealthResp online course for pharmacists and physicians: a Delphi method approach. Biomed Pharmacother 2021; 140: 111739. 34020245 10.1016/j.biopha.2021.111739

[27] Macefield R . How to specify the participant group size for usability studies: a practitioner’s GuideJUS. J Usability Stud 2009; 5: 34–45. http://uxpajournal.org/how-to-specify-the-participant-group-size-for-usability-studies-a-practitioners-guide/ (accessed April 9, 2020).

[28] Faulkner L . Beyond the five-user assumption: benefits of increased sample sizes in usability testing. Psychonomic Society Inc., Switzerland: Springer Nature, 2003. 10.3758/BF03195514 . 14587545

[29] Danial-Saad A Kuflik T Weiss PLT , et al. Usability of clinical decision support system as a facilitator for learning the assistive technology adaptation process. Disabil Rehabil Assist Technol 2016; 11: 188–194. 26203588 10.3109/17483107.2015.1070439

[30] Bangor A Kortum PT Miller JT . An empirical evaluation of the system usability scale. Int J Hum Comput Interact 2008; 24: 574–594.

[31] Orfanou K Tselios N Katsanos C . Perceived usability evaluation of learning management systems: empirical evaluation of the system usability scale. Int Rev Res Open Distance Learn 2015; 16: 227–246.

[32] Lewis JR Sauro J . The factor structure of the system usability scale. In: Lect. Notes comput. Sci. (including subser. Lect. Notes artif. Intell. Lect. Notes bioinformatics). Berlin, Heidelberg: Springer, 2009, pp.94–103. 10.1007/978-3-642-02806-9_12.

[33] Sigle S Barriga P Fernández FJC , et al. Evaluating online consumer medication information systems: comparative online usability study. JMIR MHealth UHealth 2020; 8: e16648. 32490848 10.2196/16648 PMC7301258

[34] Chu A Biancarelli D Drainoni ML , et al. Usability of learning moment: features of an E-learning tool that maximize adoption by students. West J Emerg Med 2020; 21: 78–84. 31913823 10.5811/westjem.2019.6.42657 PMC6948698

[35] Bourgeois FC Linder J Johnson SA , et al. Impact of a computerized template on antibiotic prescribing for acute respiratory infections in children and adolescents. Clin Pediatr (Phila) 2010; 49: 976–983. 20724348 10.1177/0009922810373649

[36] Mann D Knaus M McCullagh L , et al. Measures of user experience in a streptococcal pharyngitis and pneumonia clinical decision support tools. Appl Clin Inform 2014; 5: 824–835. 25298820 10.4338/ACI-2014-04-RA-0043 PMC4187097

[37] McCullagh LJJ Sofianou A Kannry J , et al. User centered clinical decision support tools: adoption across clinician training level. Appl Clin Inform 2014; 5: 1015–1025. 25589914 10.4338/ACI-2014-05-RA-0048 PMC4287678

[38] McDermott L Yardley L Little P , et al. Process evaluation of a point-of-care cluster randomised trial using a computer-delivered intervention to reduce antibiotic prescribing in primary care. BMC Health Serv Res 2014; 14: 94. 25700144 10.1186/s12913-014-0594-1 PMC4260184

[39] Rubin MA Bateman K Donnelly S , et al. Use of a personal digital assistant for managing antibiotic prescribing for outpatient respiratory tract infections in rural communities. J Am Med Inform Assoc 2006; 13: 627–634. 16929045 10.1197/jamia.M2029 PMC1656956

[40] Bangor A Kortum P Miller J . Determining what individual SUS scores mean: adding an adjective rating scale. J Usability Stud 2009; 4: 114–123. https://uxpajournal.org/determining-what-individual-sus-scores-mean-adding-an-adjective-rating-scale/ (accessed April 30, 2020).

[41] Lewis JR . The system usability scale: past, present, and future. Int J Hum Comput Interact 2018; 34: 577–590.

[42] Silva AG Simões P Santos R , et al. A scale to assess the methodological quality of studies assessing usability of electronic health products and services: Delphi study followed by validity and reliability testing. J Med Internet Res 2019; 21: e14829. 31730036 10.2196/14829 PMC6884719

