# Supplementary material for: A roadmap for the development and evaluation of the eHealthResp online course
Source: Digit Health. 2022 Mar 24;8:20552076221089088. doi: 10.1177/20552076221089088 (PMC8961349; doi:10.1177/20552076221089088)
Supplement: sj-pptx-1-dhj-10.1177_20552076221089088 - Supplemental material for A roadmap for the development and evaluation of the eHealthResp online course [file sj-pptx-1-dhj-10.1177_20552076221089088.pptx]

## Slide 1
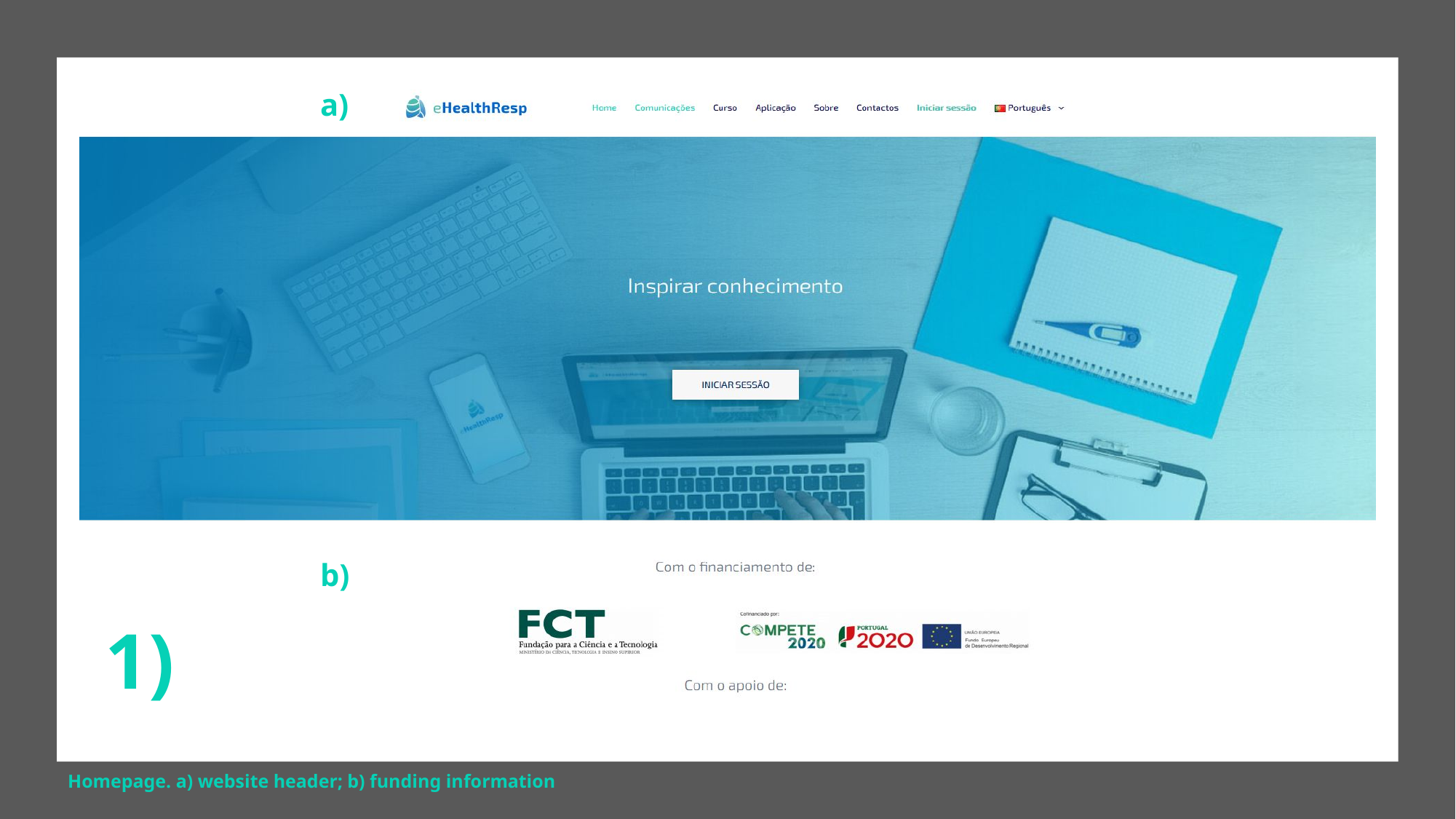

a)
b)
1)
Homepage. a) website header; b) funding information

## Slide 2
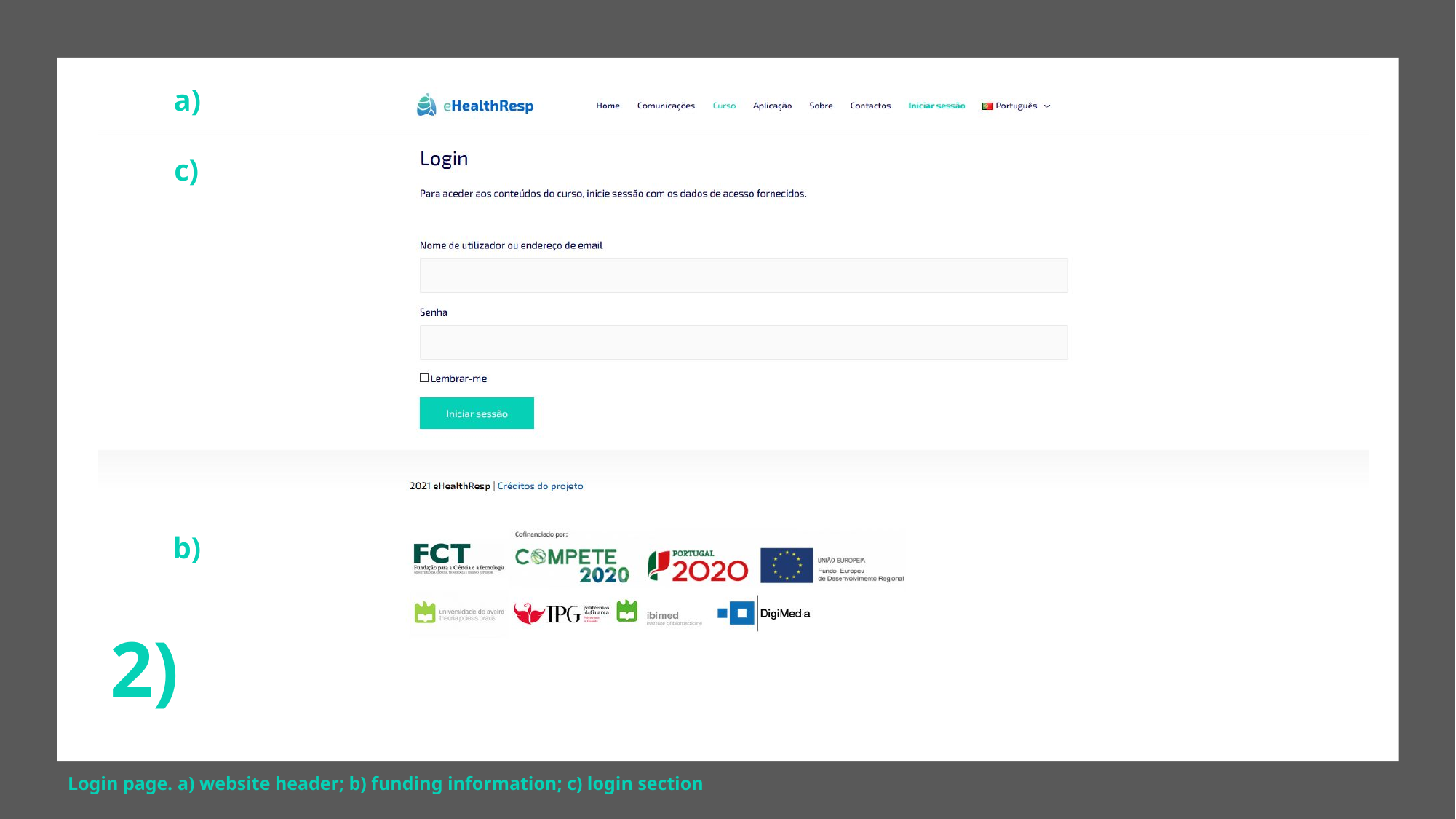

a)
c)
b)
2)
Login page. a) website header; b) funding information; c) login section

## Slide 3
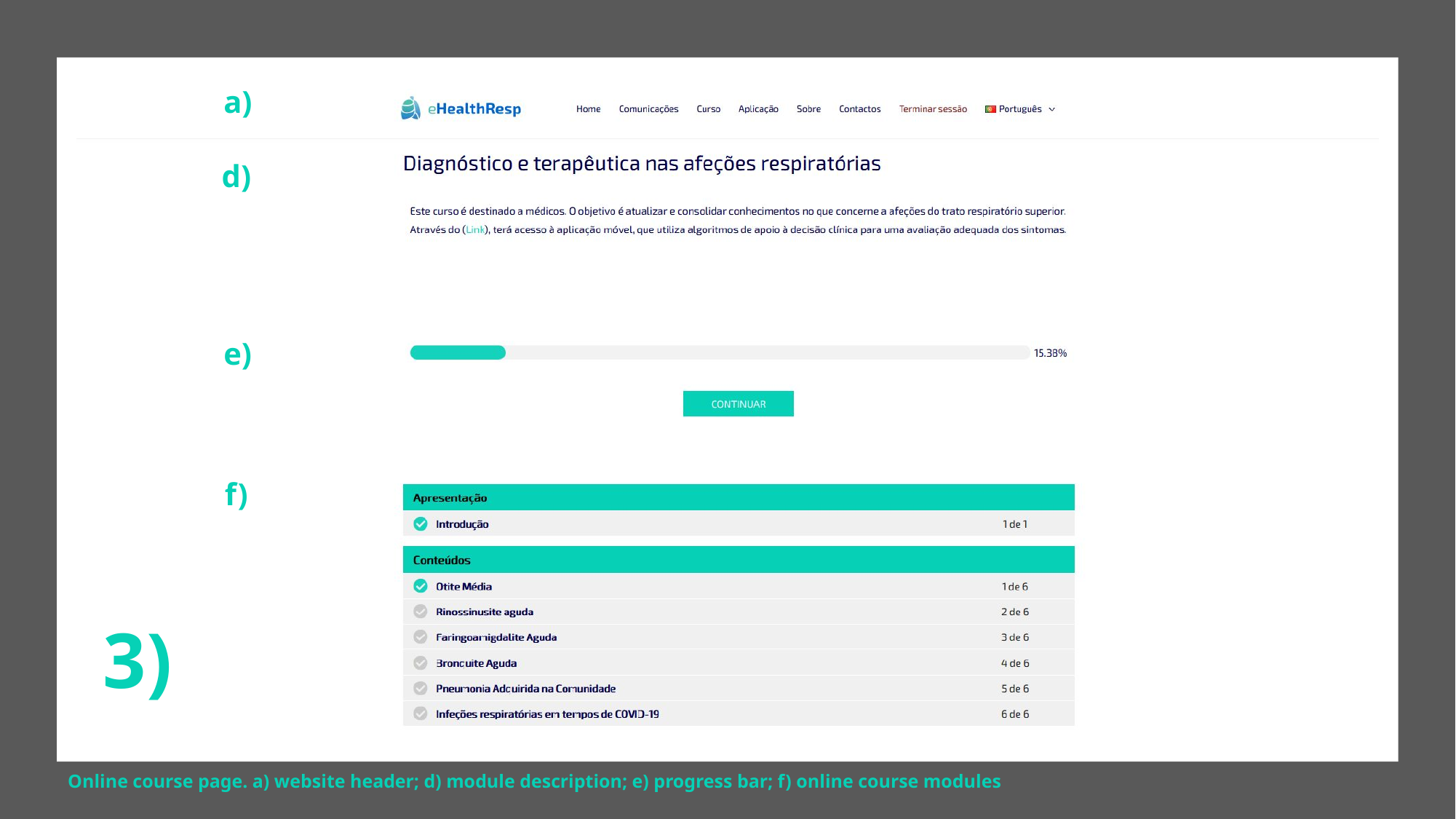

a)
d)
e)
f)
3)
Online course page. a) website header; d) module description; e) progress bar; f) online course modules

## Slide 4
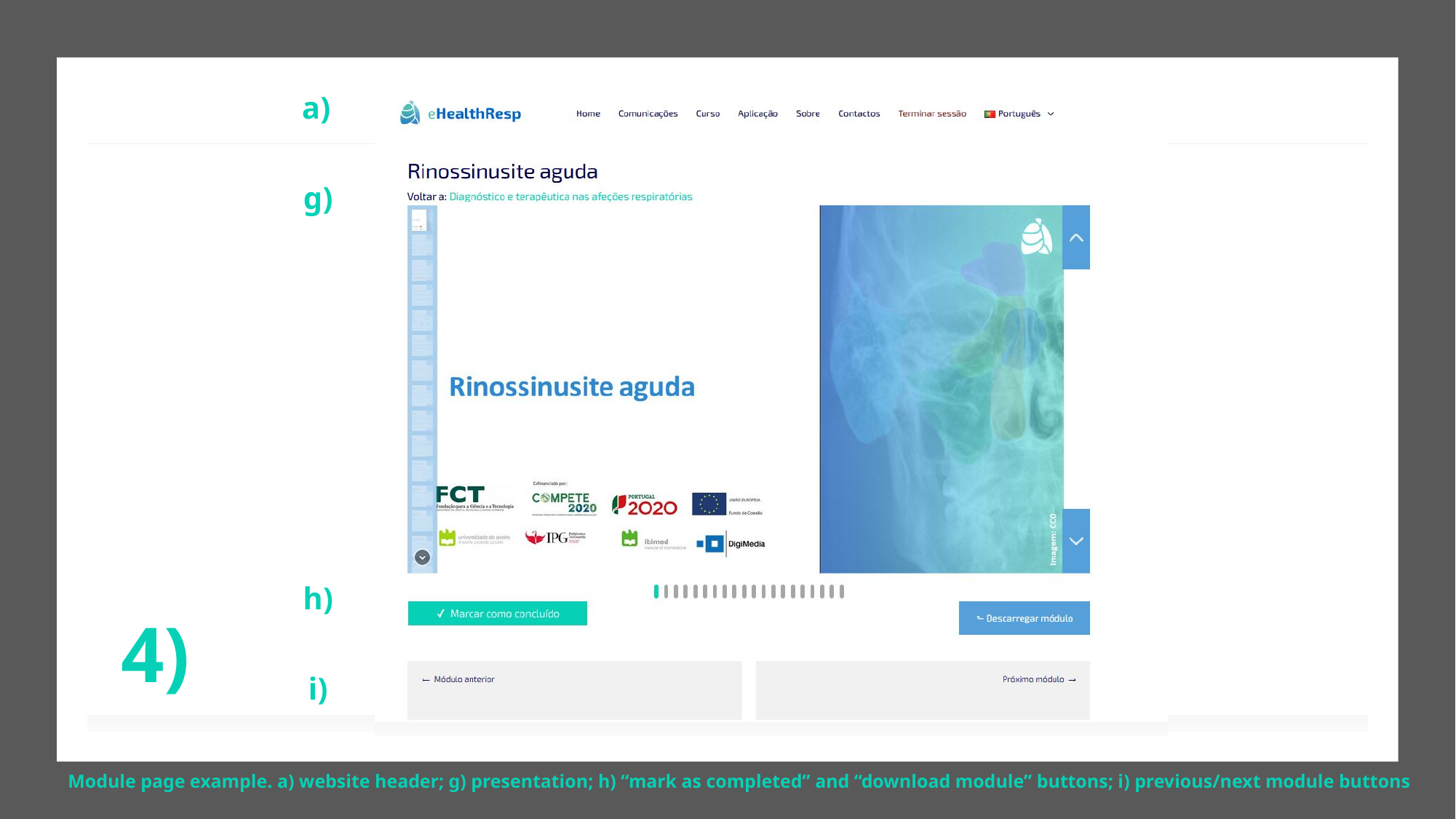

a)
g)
h)
4)
i)
Module page example. a) website header; g) presentation; h) “mark as completed” and “download module” buttons; i) previous/next module buttons

## Slide 5
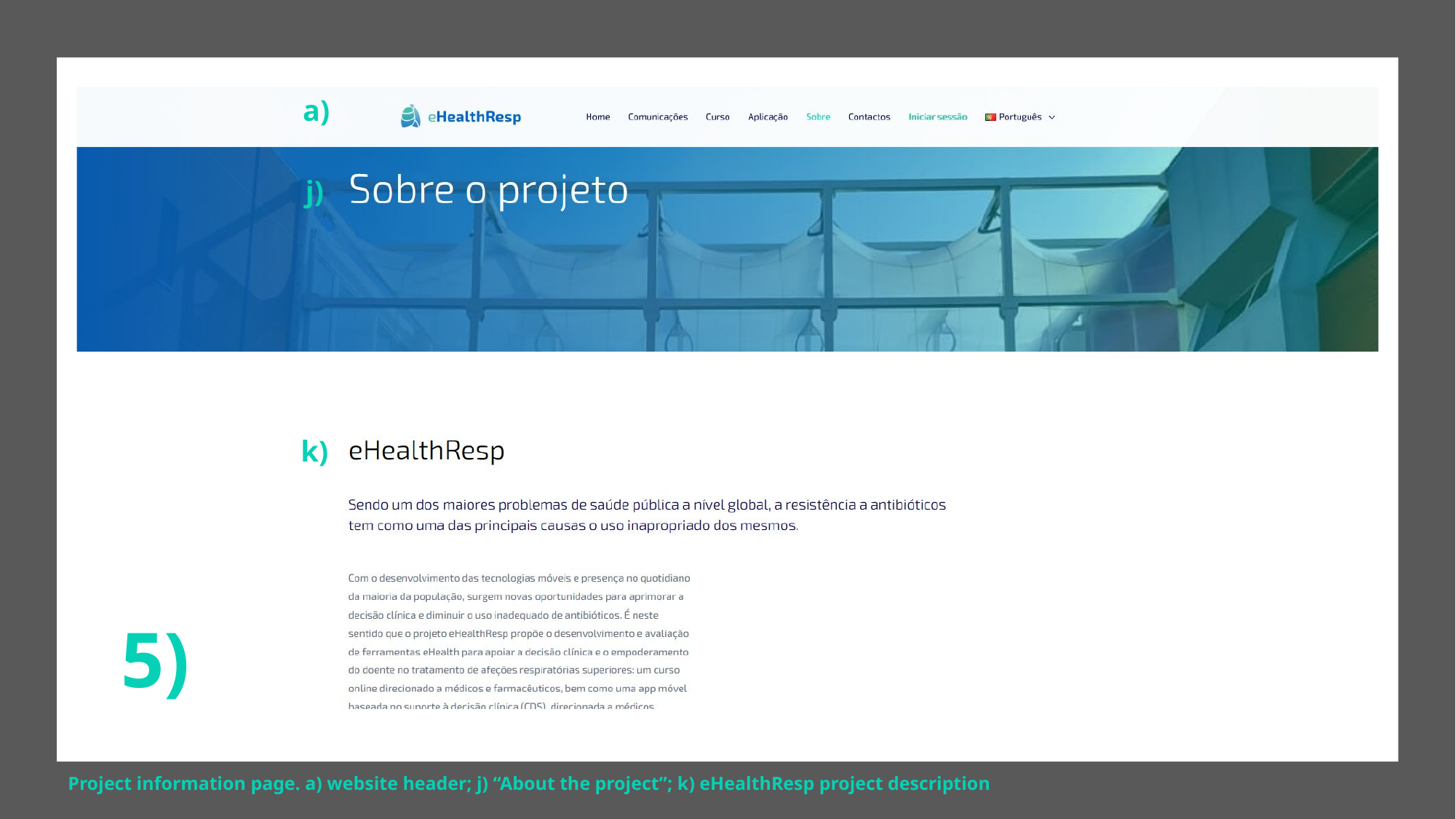

a)
j)
k)
5)
Project information page. a) website header; j) “About the project”; k) eHealthResp project description

## Slide 6
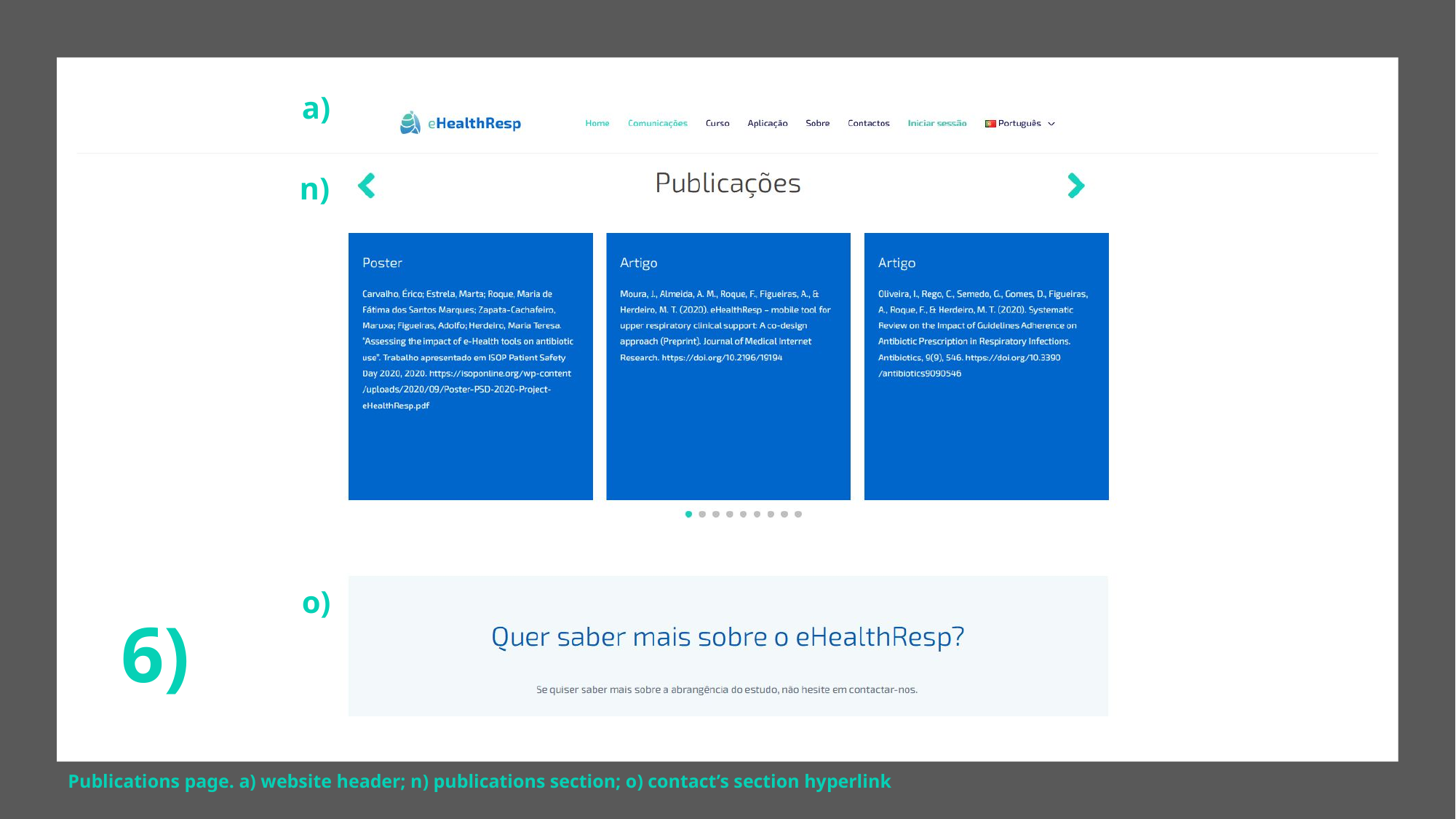

a)
n)
o)
6)
Publications page. a) website header; n) publications section; o) contact’s section hyperlink

## Slide 7
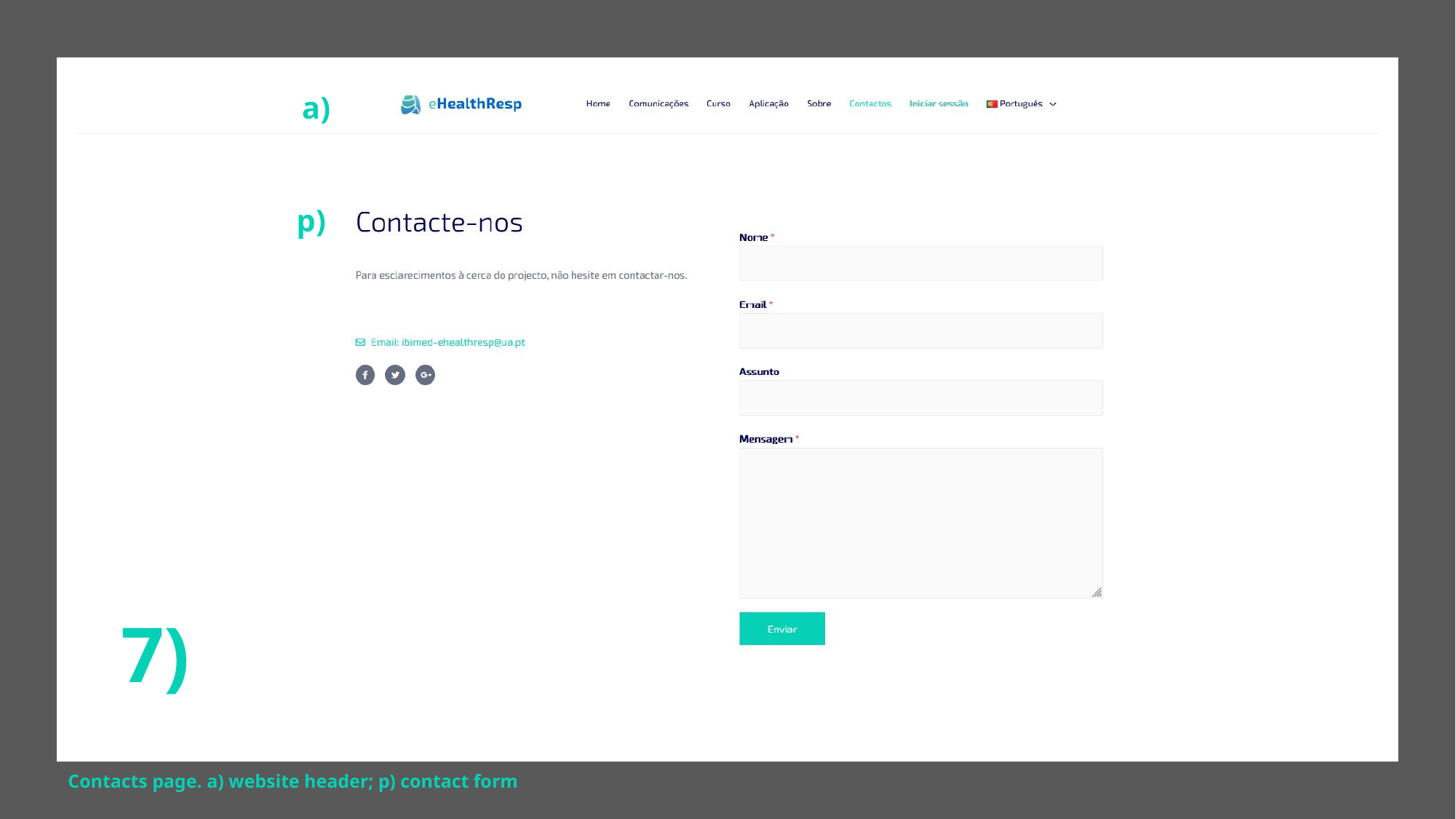

a)
p)
7)
Contacts page. a) website header; p) contact form

## Slide 8
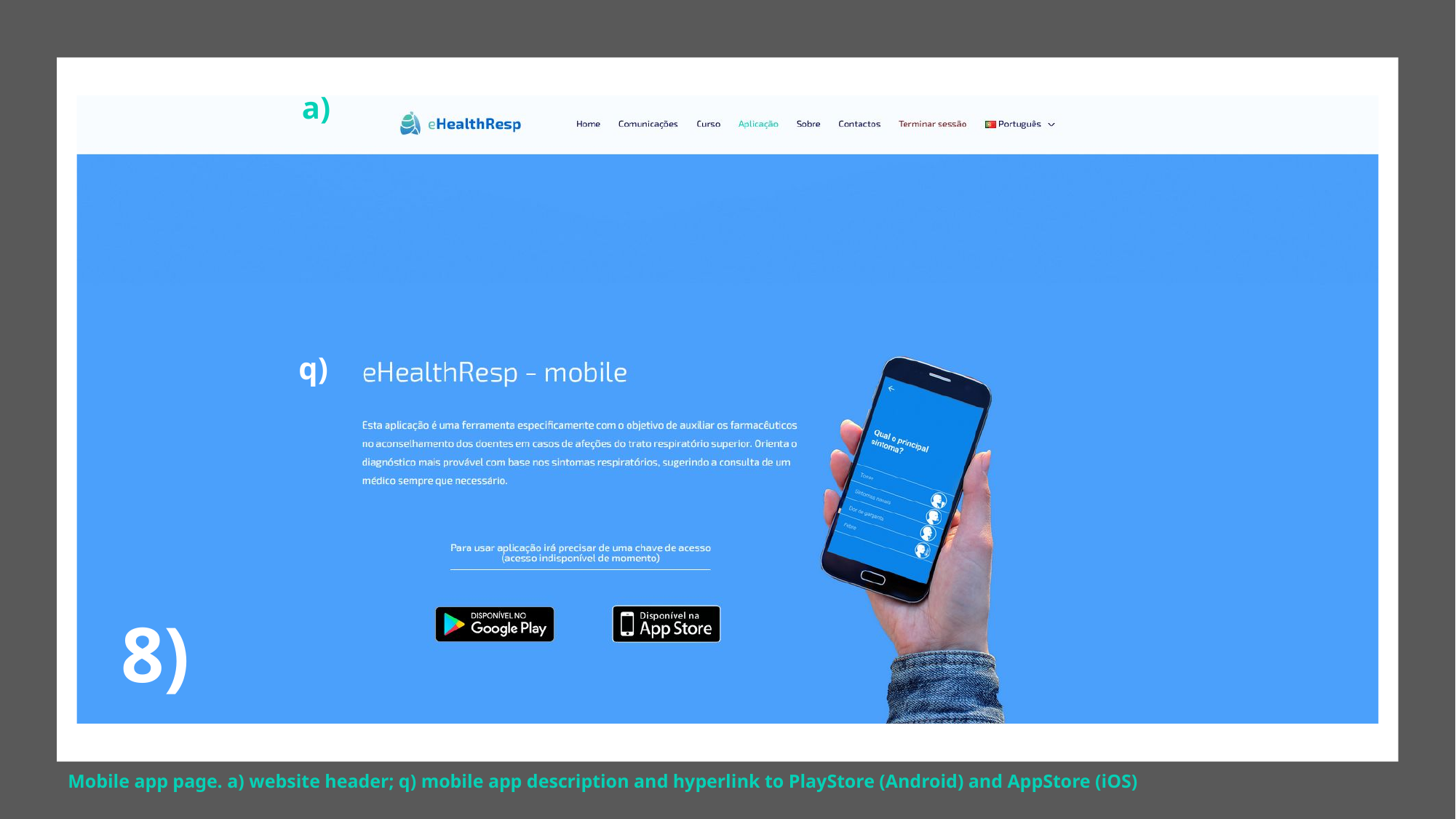

a)
q)
8)
Mobile app page. a) website header; q) mobile app description and hyperlink to PlayStore (Android) and AppStore (iOS)
